# A prospective, exploratory study of ^18^F-PSMA-1007 PET/CT in patients with medullary thyroid cancer

**DOI:** 10.1007/s00259-026-07902-6

**Published:** 2026-04-23

**Authors:** Eline C. Jager, Schelto Kruijff, Reina W. Kloet, Thera P. Links, Adrienne H. Brouwers

**Affiliations:** 1 Department of Surgery, Division of Surgical Oncology, University of Groningen, University Medical Center Groningen, Groningen, the Netherlands; 2 Department of Internal Medicine, Division of Endocrinology, University of Groningen, University Medical Center Groningen, Groningen, the Netherlands; 3 Department of Nuclear Medicine and Molecular Imaging, University of Groningen, University Medical Center Groningen, Hanzeplein 1, 9713 GZ Groningen, the Netherlands; 4 Department of Molecular Medicine and Surgery, Karolinska Institutet, Stockholm, Sweden; 5 Department of Radiology, University of Groningen, University Medical Center Groningen, Groningen, the Netherlands

**Keywords:** Medullary thyroid cancer, ^18^F-PSMA-1007 PET/CT, PSMA, ^18^F-FDG PET/CT, PET/CT imaging, Staging

## Abstract

**Purpose:**

Prostate specific membrane antigen (PSMA) is expressed in the neo-vasculature of most medullary thyroid carcinomas (MTCs) and can be imaged with PET/CT. This prospective, feasibility study evaluated the diagnostic performance of ^18^F-PSMA-1007 PET/CT in MTC patients compared to ^18^F-FDG PET/CT.

**Methods:**

Eligible patients were ≥ 1 18 years, had histopathologically confirmed MTC, elevated calcitonin/CEA, and a clinically indicated ^18^F-FDG PET/CT. All imaging ≤ 1 year prior was reviewed to establish a composite reference standard (CRS) of total tumor lesions. Analyses were performed at patient, region (neck/thorax/abdomen/bone), and lesion level. Lesions were also semi-quantified using SUVpeak.

**Results:**

In eight included patients, the CRS identified 186 tumor lesions. Patient-based sensitivity of ^18^F-PSMA-1007 PET/CT was 88% (median lesion per patient 3, range 2 – 51), while 100% for ^18^F-FDG PET/CT (lesions per patient 5, range 2 – 45). Of 32 evaluated regions, 16 (50%) were tumor positive, of which ^18^F-PSMA-1007 PET/CT and ^18^F-FDG PET/CT identified 12 (sensitivity 75%), and 14 (sensitivity 88%) correctly, respectively. ^18^F-PSMA-1007 PET/CT detected 84 lesions (sensitivity 45%) while the ^18^F-FDG PET/CT detected 85 (sensitivity 46%) of 186 tumor lesions (p = 0.893). Lesion-based semi-quantitative analysis identified a higher SUVpeak on ^18^F-PSMA-1007 (^18^F-PSMA-1007 3.37 (range 1.78 – 9.63) vs ^18^F-FDG PET/CT 2.52 (range 1.04 – 5.51) p < 0.001).

**Conclusion:**

In this exploratory, hypothesis-generating study, ^18^F-PSMA-1007 PET/CT shows modest utility in assessing disease extent in MTC patients. Its value in staging therefore seems limited when compared to existing imaging modalities. Nonetheless, high tracer uptake (exceeding the liver) in selected lesions might identify possible candidates for ^177^Lu-PSMA-617 therapy, warranting further research.

## Introduction

PET/CT imaging in patients with medullary thyroid cancer (MTC) presents significant challenges due to the low avidity and variability in MTC tracer uptake among patients [1]. This makes accurate staging at diagnosis and during follow-up difficult. However, precise diagnostic procedures are essential to assess the extent of disease and tailor treatment plans effectively [2].

Several PET tracers are currently used in clinical practice to detect MTC lesions. ^18^F-FDG PET/CT demonstrates a pooled patient-based sensitivity of 59% in MTC patients, with higher uptake in patients with aggressive tumors, particularly those with calcitonin-doubling times of less than 9 months [3–5]. However, this tracer is suboptimal for detecting indolent tumors with long calcitonin doubling-times, for which ^18^F-DOPA PET/CT has shown greater accuracy [6, 7]. Despite its advantages, ^18^F-DOPA is not as widely available compared to ^18^F-FDG. Various tracers that bind to somatostatin receptors (SSTR) are also available for MTC imaging (^68^Ga-DOTA-TOC/NOC/TATE) [1]. However, PET/CT imaging with these ^68^Ga-SSTR analogues have limited sensitivity compared to other neuroendocrine tumors, likely due to relatively low and heterogeneous SSTR expression on MTC cells [8, 9].

Determining the most optimal PET tracer is difficult to establish at baseline, as little is known about the clinical course or tumor marker secretion early in the disease [1]. Therefore, there is a need to explore additional tracers for the (re)staging of MTC patients to improve diagnostic accuracy and treatment planning.

A tracer that is worth exploring is ^18^F-PSMA, targeting prostate specific membrane antigen (PSMA). PSMA is a transmembrane protein found on the apical membrane of virtually all prostate cancer cells [10]. PSMA was thought to be very specific for prostate cancer. However, multiple studies have reported the expression of PSMA in the neo-vasculature of several other solid tumors, including MTC [11, 12]. Moreover, several case reports show incidental medullary thyroid tumors after focal uptake in the thyroid gland on a ^68^Ga-PSMA PET or ^18^F-PSMA PET for prostate cancer staging [11, 13, 14].

Multiple PSMA-directed radio-labelled tracers have been developed for PET scanning [15]. The ^18^F-PSMA-1007 tracer has demonstrated high tumor uptake, creates high contrast to background images and does not have physiological uptake in the thyroid gland [15]. Thus far, no clinical trials have studied ^18^F-labelled-PSMA in MTC patients. Furthermore, exploring this tracer in MTC patients may also be of interest for the possibility of performing PSMA-ligand peptide receptor radioligand therapy (PRRT) with ^177^Lu-PSMA-617, in case of sufficient PSMA uptake.

This feasibility study was performed to evaluate the diagnostic performance of ^18^F-PSMA-1007 PET/CT, in comparison to ^18^F-FDG PET/CT in detecting MTC lesions in patients with known biochemical disease activity.

## Methods

### Patients

This single-center, prospective, feasibility study was conducted between July 2022 and December 2024 in the University Medical Center Groningen (UMCG), a tertiary referral hospital for thyroid carcinomas in the Netherlands.

Eligible patients (≥ 18 years of age) had to have a confirmed MTC diagnosis on cytological and/or histological examination, with biochemical evidence of disease activity (meaning calcitonin and/or CEA elevated above reference values), for whom an ^18^F-FDG PET/CT was clinically indicated. Patients were excluded when they had (a history of) prostate cancer or renal cell carcinoma, were pregnant, or had recent neck surgery (< 3 months prior to inclusion). Patients were included during the primary diagnostic work-up, as well as during follow-up. All physicians treating MTC patients in our hospital were aware of the study. Potentially eligible patients were identified in the outpatient clinic or during the weekly multidisciplinary meetings (after a clinical indication for ^18^F-FDG PET/CT was set) and were subsequently screened by the primary researcher (ECJ). Patient and treatment characteristics were collected from the hospital’s electronic patient records. Calcitonin was determined using Roche Cobas 6000 immunoassay (reference values: < 10 ng/L). CEA was assessed on Abbott Laboratories immunoassay (reference values: 0.5—5 ug/L). The study was approved by the Medical Ethical Committee of the University Medical Center Groningen (2022/165) and registered at clinicaltrials.gov (NCT05534594). All patients gave written informed consent prior to participation in the study.

### Study procedures

^18^F-FDG and ^18^F-PSMA-1007 were both produced locally at the radiopharmacy of the UMCG, as has been described previously [16, 17]. For the ^18^F-PSMA-1007 PET/CT, patients were required to drink 500 ml of water in the two hours prior to their appointment and asked to void prior to scanning. For the ^18^F-FDG PET/CT, patients were required to fast during a 6-h period and required to drink 500 ml of water. Data acquisition started 55–75 min after intravenous injection of ^18^F-FDG (3 MBq/kg; range 173–285 MBq). For ^18^F-PSMA-1007 (3 MBq/kg; range 192–292 MBq), data acquisition started 40–70 min after injection. Patients were allowed to continue their medication during both study procedures. A low dose CT (ldCT) was first performed to allow attenuation and scatter correction of PET images, immediately followed by the PET acquisition. Patients were scanned from skull to the proximal femur, on either a Biograph mCT (40-slice CT) or Biograph Vision (128- slice CT) PET/CT system (both Siemens Healthineers). Per patient the same PET/CT system was used. Acquisition and reconstruction of ^18^F-PSMA PET/CT and ^18^F-FDG PET/CT are compliant with EARL1 harmonization and standardization [18]. Total scanning time covered approximately 15 min for either scan. Afterwards, patients were allowed to go home and eat and drink again.

### Visual analysis and composite reference standard

A nuclear physician (AHB) and thyroid researcher (ECJ) performed the visual assessment of the ^18^F-PSMA-1007 PET/CTs first and were blinded from other imaging modalities. Only lesions with evident and non-physiological tracer uptake above the background were regarded as MTC tumor lesions. The ^18^F-FDG PET/CT was assessed in a similar manner. Since it was neither feasible nor ethical to obtain histological evidence for each lesion on imaging, the composite reference standard (CRS) determined the total number of MTC lesions per patient (i.e. gold standard) [19]. To determine the CRS per patient, all available imaging (MRI, CT, PET/CT) < 1 year prior to the ^18^F-PSMA-1007 PET/CT (and including the ^18^F-PSMA-1007 PET/CT) were assessed by a nuclear physician (AHB) and head-neck radiologist (RK) by joint reading, and correlated with any other available cytological and histological data to determine the total presence of MTC tumor lesions [19]. Presence of lymphadenopathy on CT/MRI was based on size, morphology, density, signal intensity and location. CT/MRIs were also reviewed for presence of distant metastases, particularly sites of hematogenous metastases (liver, bone and lungs) [20–23]. Subcentimeter nodules on diagnostic CT were classified as distant metastases when demonstrating measurable contrast enhancement, internal calcifications, and/or other morphologically suspicious features, in conjunction with a clinical context of biochemical disease activity during follow-up. Since unspecific uptake in lymph nodes and bone has been reported prior for ^18^F-PSMA-1007 [11, 24], uptake in these locations that could not be confirmed on other imaging or confirmed as true tumor lesions during subsequent follow-up, were considered false positives and excluded from sensitivity calculations. These were the only instances in which the visual analysis of the index PET/CT was modified.

### Semi-Quantitative analysis

We analyzed all ^18^F-PSMA-1007 and ^18^F-FDG PET images using ACCURATE, an image analysis tool developed in-house[25]. Using a manual delineation method, abnormal lesions identified in the visual analysis were segmented after which the peak Standardized Uptake Value (SUVpeak) per lesion (corrected for bodyweight), could be determined. SUVpeak is defined as the highest average SUV of a fixed size of 1 mL within a volume of interest and was chosen because it provides a highly reproducible measure of the highest metabolic activity within a lesion, while reducing susceptibility to noise and single-voxel outliers compared with SUVmax [26]. We also determined the highest SUVpeak per patient, assessed across all quantified lesions on a patient’s ^18^F-PSMA-1007 PET/CT and ^18^F-FDG PET/CT, respectively. For the comparison of SUVpeak between ^18^F-PSMA-1007 PET/CT and ^18^F-FDG PET/CT, only concordant lesions on both scans (matched lesions) were included. Also, SUVpeak in the liver on ^18^F‑PSMA‑1007 PET/CT and ^18^F‑FDG PET/CT was measured using a 30 mm spherical volume of interest placed in a homogeneous area of the right hepatic lobe, avoiding non-parenchymal structures (vasculature, bile structures and metastases when present).

### Statistical analysis

Given that all patients exhibited increased calcitonin and CEA serum levels, any imaging modality that failed to show an abnormality was categorized as false-negative in the patient-based analysis. For the region- and lesion-based sensitivities in the visual analysis, calculated sensitivities were based on the CRS. A total of four regions per patient were assessed (neck, thorax, abdomen, bone). For the region-based sensitivity, the number of positive regions per imaging modality was divided by the total number of positive regions based on the CRS. The lesion-based sensitivity was calculated by dividing the number of positive lesions per imaging modality by the total number of positive lesions identified by the CRS. In the semi-quantitative analysis, we report median SUVpeak values for matched lesions on both ^18^F-PSMA-1007 PET/CT and ^18^F-FDG PET/CT, as well as overall median SUVpeak values for all identified lesions on ^18^F-PSMA-1007 PET/CT and ^18^F-FDG PET/CT. The highest SUVpeak on both ^18^F-PSMA-1007 PET/CT and ^18^F-FDG PET/CT was identified per patient for the patient-based analysis. For the tissue-based analysis, the median SUVpeak in all (matching) thyroid, lymph node, lung and bone lesions was determined for both scans. In the lesion-based analysis, the median SUVpeak in all (matching) lesions identified on ^18^F-PSMA-1007 PET/CT and ^18^F-FDG PET/CT was determined. Differences between scans were compared using the related-samples Wilcoxon Signed Rank Test. The correlation between tumor markers and patient-based SUVpeak was evaluated with Pearson’s Correlation. All statistical analyses were performed in IBM SPSS Statistics Version 29. P-values < 0.05 were considered statistically significant.

## Results

### Patients

Eight patients with medullary thyroid carcinoma were included (5 females and 3 males) (Table 1). One patient had a germline *RET* mutation consistent with Multiple Endocrine Neoplasia type 2A. The median age at time of inclusion was 57 years (range 24–76). One patient was included in the primary diagnostic work-up, while the other seven were already in follow-up and received ^18^F-FDG PET/CT imaging to assess the extension of the disease, and/or the degree of disease progression. The time since diagnosis, i.e. first cytological/histological evidence of MTC, varied widely with a median of 57 months (range 2–383). All patients had elevated calcitonin and CEA, consistent with the inclusion criterium. Median calcitonin and CEA concentrations were 1592 ng/L (range 58–75,440) and 39 ug/L (range 5–612) when the scans were performed.

All eight patients had ^18^F-PSMA-1007 PET/CT and ^18^F-FDG PET/CT imaging, as per the study protocol. The scans were performed within 8 weeks of each other (median time between scans was 33 days, range 1–51). All other imaging < 1 year prior to the ^18^F-PSMA-1007 PET/CT was also assessed to determine CRS. In the eight patients, four patients had a prior ^18^F-DOPA PET/CT, and six patients had prior CT/MRI imaging.

**Table 1:** Patient characteristics

Clinical characteristics										
**Patient**	**Age**	**Sex**	**Type MTC**	**Diag/FU**	**Calcitonin ng/L**	**CEA** **ug/L**	**T**	**N**	**M**	**Location of distant metastases**
1	72	Male	Spor	FU	1845	12	T2	N1b	M1	Lungs
2	24	Female	Spor	FU	75,440	612	TX	N1b	M1	Mediastinum, lungs, bone
3	30	Male	Spor	FU	4583	40	T2	N1b	M1	Lungs
4	37	Female	Spor	FU	1466	38	T3a	N1b	M0	
5	76	Male	Spor	FU	562	13	T1b	N1b	M1	Lungs
6	57	Female	MEN2A	FU	1717	129	TX	NX	M1	Bone
7	57	Female	Spor	Diag	58	5	T1a	N1b	M0	
8	74	Female	Spor	FU	1082	78	T2	N1a	M1	Mediastinum, bone

Abbreviations: MTC = medullary thyroid cancer, diag = diagnosis, FU = follow-up, M = male, F = female, Spor = sporadic medullary thyroid cancer, MEN2A = Multiple Endocrine Neoplasia Type 2 A, CRS = composite reference standard

### Visual analysis

#### Patient-based analysis

**Fig. 1:**
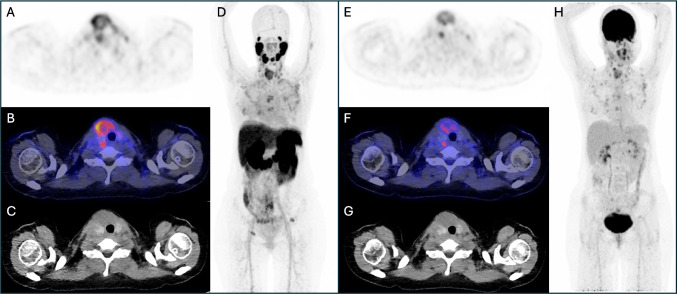
Images of ^18^F-PSMA-1007 PET/CT (left) and ^18^F-FDG PET/CT (right) in a medullary thyroid cancer patient with extensive distant metastatic disease in the thyroid, locoregional lymph nodes, lungs and bone. A-C: transverse view at the level of the primary thyroid tumor on ^18^F-PSMA-1007 PET/CT, D: maximum intensity projection (MIP) of ^18^F-PSMA-1007 PET/CT, E–G: transverse view at the level of the primary thyroid tumor on ^18^F-FDG PET/CT (same patient), H: MIP of ^18^F-FDG PET/CT. The authors affirm that the human research participant provided informed consent for publication of the images in this figure

A total of 186 distinct tumor lesions based on the CRS per patient were identified (see Table 2). ^18^F-PSMA-1007 PET/CT generated images that were straightforward to interpret (Fig. 1) and identified tumor lesions in seven patients (sensitivity 88%). One patient had a false negative ^18^F-PSMA-1007 PET/CT. The median number of tumor lesions per patient (lpp) was 3 (range 2–51). In contrast, the ^18^F-FDG PET/CT detected lesions in all eight patients (sensitivity 100%, median lpp 5 range 2—45). Of seven patients with diagnostic imaging (CT/MRI), all were positive (sensitivity 100%, median lpp 12 range 1–72). Four patients had a prior ^18^F-DOPA PET/CT which showed tumor lesions in all four (sensitivity 100%, median lpp 4 range 2–36).

**Table 2:** Visual analysis—CRS per patient and in total, and the number of lesions per patient per imaging modality

Patient	CRS (n = 8)	^18^F-PSMA (n = 8)	^18^F-FDG (n = 8)	MRI/CT (n = 7)		^18^F-DOPA (n = 4)
1	25	13	13	CT head/neck, thorax, abdomen	23	-
2	82	51	45	CT head/neck, thorax; MRI spinal column	72	-
3	13	0	4	CT head/neck, thorax	12	6
4	10	10	6	CT head/neck, thorax	9	2
5	13	3	2	CT head/neck	13	-
6	38	3	11		-	36
7	3	2	2	CT thorax; MRI head/neck	3	2
8	2	2	2	MRI pelvis	1	
*Sum*	*186*	*84*	*85*		*88*	*43*

Abbreviations: CRS = composite reference standard: total number of distinct lesions based on all available imaging technique per patient

#### Region-based analysis

A total of 32 regions in these eight patients were evaluated, and 16 (50%) were considered positive for tumor based on the CRS. While 12 out of these 16 regions were positive on ^18^F-PSMA-1007 PET/CTs (sensitivity 75%), ^18^F-FDG PET/CT correctly identified 14 out of the 16 regions (sensitivity 88%) (Table 3). ^18^F-PSMA-1007 PET/CT did not identify more tumor lesions per region than ^18^F-FDG PET/CT (neck p = 0.357, thorax p = 0.414, abdomen p = 1.000, bone p = 0.655). Since the extent of diagnostic imaging varied in the six patients with CT/MRI (Table 2), not all regions in the seven patients were assessed. A total of 16 regions were assessed with CT/MRI and 12 were positive for tumor lesions on CT/MRI (Table 3). The corresponding CRS of the 16 assessed regions was 13, giving a region-based sensitivity of 92% for CT/MRI. In the four patients with ^18^F-DOPA PET/CT, a total of 16 regions were assessed of which six were positive. The CRS considered seven positive regions in these patients, resulting in 86% region-based sensitivity for ^18^F-DOPA PET/CT.

**Table 3:** Visual Analysis—region-based and lesion-based analysis for the CRS and each individual imaging modality

	**CRS**	^**18**^**F-PSMA PET/CT**	^**18**^**F-FDG PET/CT**	**CT/MRI**^1^	^**18**^**F-DOPA PET/CT**^2^
Number of patients	n = 8	n = 8	n = 8	n = 7	n = 4
Positive regions	16 (100%)	12 (75%)	14 (88%)	12 (92%)	6 (86%)
Head and neck	6	5	6	5	2
Thorax	6	4	5	5	2
Abdomen	1	0	0	0	1
Bone	3	3	3	2	1
Positive lesions	186 (100%)	84 (45%)	85 (46%)	132 (90%)	46 (72%)
Head and neck	40	33	28	37	7
Thorax	67	27	32	63	3
Abdomen	5	0	0	0	5
Bone	74	24	25	32	31

^1^For the 7 patients with some form of diagnostic imaging (CT/MRI), a total of 16 regions were assessed and reported sensitivities are relative to the CRS within these regions. ^2^For the four patients with ^18^F-DOPA PET/CT, a total of 16 lesions were assessed and reported sensitivities are relative to the CRS within these regions. Abbreviations: CRS = composite reference standard

#### Lesion-based analysis

No significant difference was detected in the lesion-based sensitivity of ^18^F-PSMA-1007 PET/CT and ^18^F-FDG PET/CT (p = 0.893). ^18^F-PSMA-1007 PET/CT detected 84 (sensitivity 45%) of the total of 186 tumor lesions while the ^18^F-FDG PET/CT detected 85 (sensitivity 46%) tumor lesions (Table 3). Patient 2 had the most lesions on both imaging modalities, see Fig. 1. In the seven patients with diagnostic imaging, 132 lesions were identified (sensitivity 90%). CT detected multiple intrapulmonary nodules in patient 3 and 5, which were still too small for any PET/CT imaging to detect. ^18^F-DOPA PET/CT identified 46 tumor lesions in the total of four patients (sensitivity 72%).

Patient 1 and 7 each had one bone lesion on the ^18^F-PSMA-1007 PET/CT that could not be confirmed on any other available imaging modality, including the corresponding low-dose CT. Neither did these patients have other bone metastases. In addition, low-grade uptake of ^18^F-PSMA-1007 was seen in cervical lymph nodes of patient 7 and 8, which could not be correlated with any other imaging modality, pathology results or follow-up scans. Patients 1, 3 and 5 had multiple sub-centimeter pulmonary nodules on CT imaging only (under the detection limit of PET/CT), which resulted in a higher CRS and lowered the sensitivity rates of both PET modalities.

### Semi-Quantitative analysis

Not all lesions identified in the visual analysis could be accurately delineated due to small size or adjacency to structures with physiological uptake, resulting in a lower number of lesions suitable for the semi-quantitative analysis. Of the total of 84 lesions identified in the visual analysis of all ^18^F-PSMA-1007 PET/CTs, 69 could be delineated to allow semi-quantitative analyses. Of 85 lesions on ^18^F-FDG PET/CTs, 66 were suitable for segmentation. In total, 45 matching lesions could be delineated on both ^18^F-PSMA-1007 PET/CT and ^18^F-FDG PET/CT scans.

#### Patient-based analysis

We identified no significant difference in SUVpeak between ^18^F-PSMA-1007 positive lesions (median SUVpeak 3.71 range 2.45–9.63) and ^18^F-FDG positive lesions (median 3.61 range 1.88–5.51) in the patient-based analysis (p = 0.398). The median SUVpeak of normal liver parenchyma was 1,66 (range 1,27–9,76) on ^18^F-PSMA-1007 PET/CT and 2,83 (range 1,92–3,35) on ^18^F-FDG PET/CT.

#### Tissue-based analysis

SUVpeak was higher for ^18^F-PSMA-1007 in lymph nodes and bone lesions compared to ^18^F-FDG (p = 0.010, p = 0.005, respectively). Median SUVpeak for matching lesions are reported in Table 4. Overall, median SUVpeak in lymph node metastases was 2.81 (range 1.18–9.63) on ^18^F-PSMA-1007 PET/CT, and 2.56 (range 1.04–4.87) on ^18^F-FDG PET/CT. In lung metastases, a median SUVpeak of 3.40 (range 2.16–4.46) and 2.40 (range 1.80–4.20) was identified on ^18^F-PSMA-1007 PET/CT and ^18^F-FDG PET/CT, respectively (p = 0.345). Bone metastases had a median SUVpeak of 2.51 (range 1.60–4.85) and 2.55 (range 1.19–3.85), on ^18^F-PSMA-1007 PET/CT and ^18^F-FDG PET/CT, respectively. Median SUVpeak in thyroid tumor lesions was 5.29 (range 3.54–7.04) on ^18^F-PSMA-1007 PET/CT (n = 2 patients). In only one patient a thyroid lesion was visible on ^18^F-FDG PET/CT with a SUVpeak of 5.51.

**Table 4:** Semi-quantitative analysis—tissue-based analysis of matched lesions with SUVpeak

	**SUVpeak, median (range)**			**Lesions segmented, no**		
	^**18**^**F-PSMA PET/CT**	^**18**^**F-FDG PET/CT**	**P-value**^**2**^	^**18**^**F-PSMA PET/CT**	^**18**^**F-FDG PET/CT**	**Matching lesions**
Thyroid	7.04 (NA)^1^	5.51 (NA)^1^	-	2	1	1
Lymph nodes	3.37 (1.78–9.63)	2.85 (1.04–4.87)	0.010	37	35	25
Lungs	3.40 (2.16–4.46)	2.40 (1.80–4.20)	0.345	6	6	6
Bone	3.35 (1.81–4.85)	2.36 (1.19–3.85)	0.005	24	24	13

^1^ Since only 1 thyroid lesion matched on both scans, no range and p-value could be reported. ^2^ Difference in median SUVpeak per tissue-type, assessed across matching lesions

#### Lesion-based analysis

In the lesion-based analysis, SUVpeak was significantly higher on ^18^F-PSMA-1007 PET/CT in the subset of 45 matching lesions detected on both scans (^18^F-PSMA-1007 PET/CT 3.37 (range 1.78–9.63) vs ^18^F-FDG PET/CT 2.52 (range 1.04–5.51) p < 0.001). Assessed overall, a median SUVpeak of 2.94 (range 1.18–9.63) was identified in all lesions on ^18^F-PSMA-1007 PET/CTs. In all ^18^F-FDG PET/CT lesions, median SUVpeak was 2.54 (range 1.04–5.51). SUVpeak exceeded liver SUVpeak in 22 of 69 quantified lesions (32%) on ^18^F-PSMA-1007 PET/CT (13/22 [59%] by a factor ≥ 1.5) and in 22 of 66 lesions (33%) on ^18^F-FDG PET/CT (5/22 [23%] by a factor ≥ 1.5).

### Tumor markers

No significant correlations were identified between calcitonin and CEA and patient-based SUV-peak on ^18^F-PSMA-1007 PET/CT or ^18^F-FDG PET/CT. Calcitonin doubling times also did not correlate with SUVpeak on either scan in this small cohort.

## Discussion

We report, for the first time, an exploratory, prospective evaluation of ^18^F-PSMA-1007 PET/CT in eight patients with MTC. This study identified modest sensitivity rates of ^18^F-PSMA-1007 PET/CT for MTC tumor lesions. While patient-based sensitivity was high (88%), region- and lesion-based sensitivities were slightly lower (75% and 45%) when compared with sensitivity rates of 100%, 88% and 46% of ^18^F-FDG PET/CTs, respectively. Median ^18^F-PSMA-1007 tracer uptake varied considerably between patients and was higher when compared to that of ^18^F-FDG, particularly in lymph nodes and bone lesions.

In this small group of MTC patients, ^18^F-PSMA-1007 PET/CT performed reasonably well, detecting MTC lesions in seven out of eight patients and even outperforming ^18^F-FDG PET/CT in some patients. The region- and lesion-based sensitivity rates of ^18^F-PSMA-1007 PET/CT were low, particularly when compared to the sensitivity observed in prostate cancer (> 90%). This discrepancy is likely due to the high PSMA expression on the epithelial surface of prostate cancer cells and their reduced dependence on neo-angiogenesis for effective imaging, which MTC typically requires [12, 27]. The proportion of detected lesions on ^18^F-PSMA-1007 PET/CT varied between patients, likely reflecting variability in PSMA expression in tumor tissues, allowing sufficient binding and visualization on PET/CT imaging in some patients and limited performance in others*.*

PSMA-based PET/CT imaging has been evaluated in other malignant diseases with PSMA expression in the neovasculature. De Vries et al. performed ^68^Ga-PSMA PET/CT in 5 patients with radio-iodine refractory thyroid cancer and demonstrated tracer uptake in all patients [28]. In their study, in which they compared median SUVmax (maximum pixel in a region-of-interest) instead of SUVpeak, the reported median SUVmax was 3.59 (range 2.90–5.13) for lymph node metastases and 4.06 (range 0.85—10.56) for distant metastatic lesions, showing considerable heterogeneity in tracer uptake [28]. These findings are in the same range as the respective reported median SUVpeak in our study. In 33 patients with non-small cell lung cancer, ^68^Ga-PSMA PET/CT was highly sensitive for primary lung lesions (sensitivity 97%) and superior to ^18^F-FDG PET/CT in detecting brain metastases [29]. For breast cancer, a lesion-based sensitivity of 84% has been reported with ^68^Ga-PSMA PET/CT in 21 patients [30].

Relative to already available imaging techniques, ^18^F-PSMA-1007 PET/CT was of limited additional value in identifying total tumor load in our study. Limitations, particularly non-specific bone uptake, have been previously reported for ^18^F-PSMA-1007 PET/CT imaging in other diseases and may lead to inaccurate staging [11, 24]. In addition, PSMA-based radiotracers are not very specific, since PSMA expression in the neovasculature has been reported for various other benign and malignant diseases [11, 31]. Finally, physiological uptake in the liver may impede accurate localization of liver metastases while salivary gland uptake challenges the distinction from neighboring lymph node metastases. Concerning limitations of the study, we realize that the included cohort is small, which is related to the rareness of this tumor type. This impedes generalization of the results and more advanced comparative quantitative analyses. For example, due to three patients with multiple sub-centimeter lung nodules, the sensitivity of both PET/CT scans was negatively affected. In addition, use of a CRS – while ethically justified—may have introduced some bias as lesions could not always be pathologically confirmed. The cohort was also heterogeneous, comprising patients included at different stages of care: a minority during the primary diagnostic phase, most during follow-up, and with variations in biochemical disease activity.

Potentially, ^18^F-PSMA-1007 PET/CT may serve as a theranostic modality to identify patients with high uptake, who may be suitable candidates for PSMA-ligand PRRT with ^177^Lu-PSMA-617. This treatment has shown to be safe and highly effective in patients with metastatic castration-resistant prostate cancer, with a reported objective response rate of 82% after four cycles [32]. In the study of Hofman et al., suitable patients were selected when SUVmax in tumor lesions is at least 1.5 times higher than normal physiological uptake of the liver [32]. The preliminary study of De Vries et al. treated two RAI-DTC patients with ^177^Lu-PSMA-617 after screening with ^68^Ga-PSMA PET/CT. The two treated patients had at least one lesion with SUVmax > 4. While one patient seemed to have some beneficial treatment effect, the small sample limited unbiased conclusions [28]. So far, no studies have explored ^177^Lu-PSMA-617 treatment in MTC patients. Since PSMA uptake in tumor lesions was only modest and quite heterogeneous in this small series, to allow such a theranostic trial, more data on the general performance of ^18^F-PSMA PET/CT in a larger cohort of MTC patients would first be required. In such a study, tracer uptake in tumor lesions, relative to liver uptake and relative to somatostatin receptor expression could also be explored to guide potential candidate selection.

In conclusion, we show that ^18^F-PSMA-1007 PET/CT is feasible in patients with MTC. In this small exploratory, hypothesis-generating cohort, it shows modest utility in assessing disease extent in MTC patients. However, its value in staging seems limited when compared to existing imaging modalities. The detection of high ^18^F-PSMA-1007 tracer uptake (exceeding liver uptake in selected patients) might identify possible candidates for ^177^Lu-PSMA-617 therapy. However, this warrants further research.

## Data Availability

The datasets generated during and/or analyzed during the current study are available from the corresponding author on reasonable request.
